# Transperineal ultrasound beyond prostate biopsy: pictorial essay

**DOI:** 10.1590/0100-3984.2024.0071-en

**Published:** 2025-03-11

**Authors:** Gabriel Franchi De Santi, Bernardo Oliveira Pacheco, Guilherme Cayres Mariotti, Denis Szejnfeld, Thiago Franchi Nunes

**Affiliations:** 1 Escola Paulista de Medicina da Universidade Federal de São Paulo (EPM-Unifesp), São Paulo, SP, Brazil; 2 Hospital Israelita Albert Einstein (HIAE), São Paulo, SP, Brazil; 3 Interventix, Campo Grande, MS, Brazil

**Keywords:** Perineum/diagnostic imaging, Biopsy, Drainage, Radiotherapy, Hydrogels, Ultrasonography., Períneo/diagnóstico por imagem, Biópsia, Drenagem, Radioterapia, Hidrogéis, Ultrassonografia.

## Abstract

For ultrasound-guided prostate biopsy, a transperineal approach is emerging as a superior alternative to the transrectal approach because the former is associated with a lower risk of infection. This pictorial essay aims to highlight the broader applications of transperineal ultrasound (i.e., those beyond prostate biopsy). We demonstrate various diagnostic and therapeutic uses of transperineal ultrasound, including lymph node biopsies, abscess drainage, hydrogel spacer placement for radiotherapy, and penile biopsies. Details of the transperineal approach, including patient positioning and preparation, are described. In addition, the effectiveness and safety of the method are demonstrated. Our results underscore the versatility of transperineal ultrasound and its potential to enhance clinical practice, demonstrating its importance as a minimally invasive technique with significant clinical benefits in various medical contexts.

## Abstract (pt)

A biópsia de próstata guiada por ultrassom transperineal está se destacando como uma alternativa superior à biópsia guiada por ultrassom transretal, em razão do seu menor risco de infecção. Este ensaio iconográfico tem como objetivo destacar as aplicações mais amplas do ultrassom transperineal, além das biópsias de próstata. O estudo demonstra vários usos diagnósticos e terapêuticos do ultrassom transperineal, incluindo biópsias de linfonodos, drenagem de abscessos, colocação de afastadores de hidrogel para radioterapia e biópsias de lesões penianas. O procedimento detalhado, o posicionamento e a preparação do paciente são descritos, enfatizando a eficácia e a segurança do método. Os resultados ressaltam a versatilidade do ultrassom transperineal e seu potencial para aprimorar práticas clínicas, demonstrando sua importância como uma técnica minimamente invasiva, com benefícios clínicos significativos em diversos contextos médicos.

## INTRODUCTION

Ultrasound-guided transrectal prostate biopsy is the most common method of prostate biopsy worldwide^**(1)**^. However, this procedure has been associated with a significant risk of sepsis^**(2)**^. Alternatively, ultrasound-guided transperineal prostate biopsy, in which the ultrasound probe is inserted into the rectum and biopsy samples are collected through the perineum, is considered a “clean” procedure, whereas ultrasound-guided transrectal prostate biopsy is considered a “contaminated” procedure. Traditionally, transperineal prostate biopsy is performed under local anesthesia and sedation, with a sampling pattern ranging from 20 to 45 biopsy cores^**(3)**^. The transperineal approach has been shown to significantly reduce postprocedural infection rates^**(4-10)**^.

This pictorial essay aims to demonstrate the comprehensive interventional technique of an ultrasound-guided transperineal approach, highlighting other diagnostic and therapeutic applications beyond prostate biopsy. The importance and applicability of transperineal access are emphasized as a refined technique and an effective approach in diagnosis and treatment in diverse clinical contexts.

## PATIENT POSITIONING AND PREPARATION

**Figure 1:**
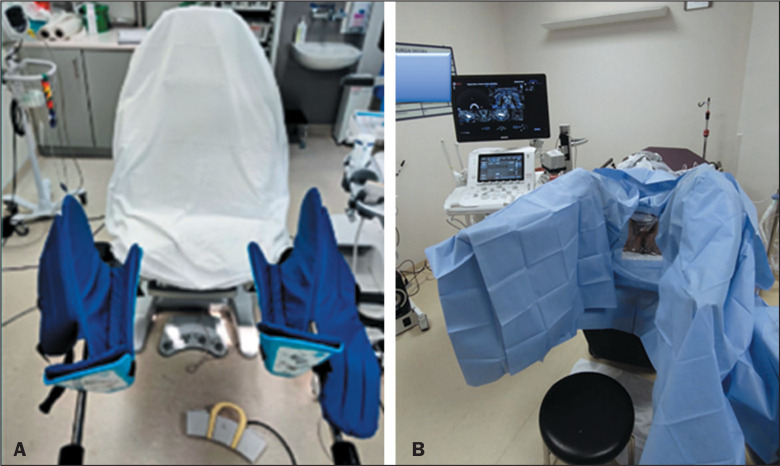
A: Stretcher with leg rests for patient positioning. The stretcher has movable stirrups that allow adequate positioning, providing comfort to the patient and ideal access for the biopsy. B: Patient in a modified lithotomy position on the stretcher.

The patient is placed in a modified lithotomy position, with the legs elevated, abducted, and supported in stirrups; the buttocks should project slightly beyond the lower edge of the table (Figure 1). After the patient has been positioned, the scrotum is fixed cranially. Relaxation of the perineal muscles is crucial to allow movement of the probe into the rectum and passage of the introducer needle through the levator muscles. External rotation of the hips assists in relaxing the muscles of the upper thighs.

**Figure 2:**
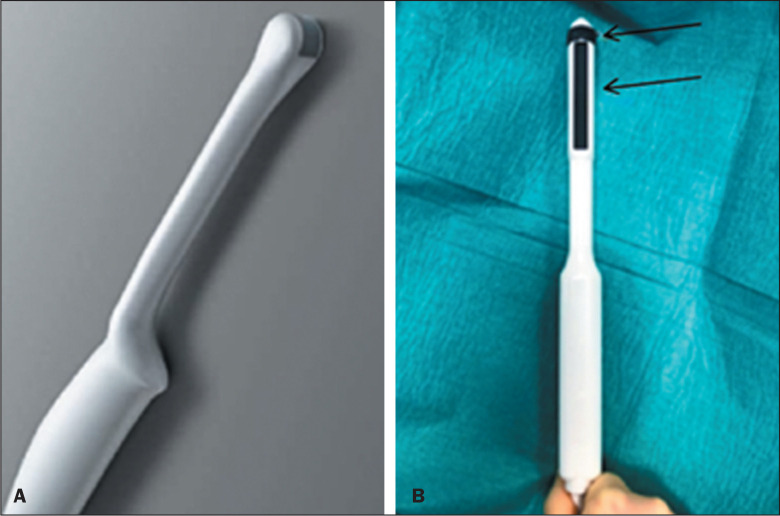
A: Axial endocavitary transducer used. B: Biplanar endocavitary transducer (upper arrow - axial; lower arrow - sagittal).

The perineum can be shaved, and skin antisepsis is achieved with chlorhexidine, after which sterile drapes are put in place. A biplanar transrectal probe (Figure 2) can be guided by biopsy maps and by fusion with magnetic resonance imaging (MRI). The procedure can now be performed under sedation and local anesthesia, allowing it to occur in an outpatient setting and significantly reducing costs^**(5,6)**^.

## ULTRASOUND-GUIDED TRANSPERINEAL INTERVENTIONS

To explore applications of the transperineal approach beyond prostate biopsy, we selected cases of patients undergoing the following: i) drainage of a pelvic abscess; ii) biopsy of a lymph node in the pelvic region; iii) placement of a hydrogel spacer for radiotherapy; iv) biopsy of a perineal lesion.

### Drainage of a pelvic abscess

**Figure 3:**
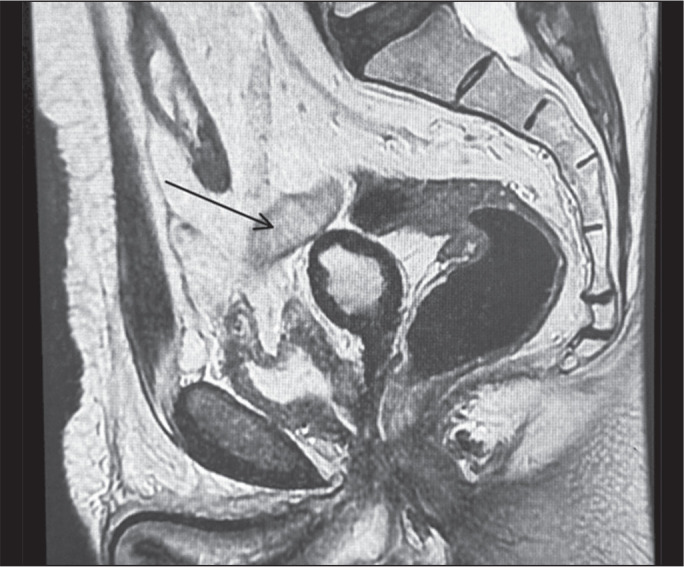
Sagittal T2-weighted MRI slice showing a collection (arrow) that appeared after robotic prostatectomy.

**Figure 4:**
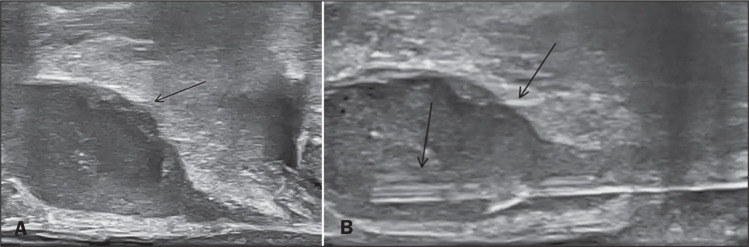
A: Ultrasound showing the same collection seen on MRI. B: Drainage performed via the transperineal approach. Note the 18-gauge Chiba needle (lower arrow), used for aspiration, inside the collection (upper arrow).

A 68-year-old male patient presented with severe pelvic pain and fever after robotic prostatectomy. Clinical examination and MRI indicated an abscess in the prostate cavity (Figure 3). The collection was also identified by ultrasound (Figure 4A). Drainage was performed by aspiration with an 18-gauge Chiba needle, under ultrasound guidance, via the transperineal route, and was successfully completed. The symptoms improved (Figure 4B), and the patient was discharged early the following day.

For the drainage of a pelvic abscess, the use of the transperineal route, rather than the transrectal route, can be justified for several reasons, including the fact a transperineal approach reduces the risk of contamination because, unlike the transrectal route, the transperineal route does not pass through the rectum (which is highly colonized by bacteria), thus decreasing the risk of contamination and secondary infection. Another reason to use a transperineal approach for drainage is that it allows better access to deep collections (i.e., direct access to collections that are located deep in the pelvis, especially in postsurgical patients, such as those recovering from prostatectomy). Anatomical alterations due to surgery can complicate transrectal access, which can be less safe because of those alterations, adhesions, or proximity to important vascular or nervous structures. Transperineal access can bypass those complications, offering a safer route, especially in some postoperative scenarios.

In short, the transperineal route was chosen in this case to avoid contamination and to offer a safer and more direct route to pelvic collections, especially in patients with anatomical alterations from previous surgery.

### Pelvic lymph node biopsy

**Figure 5:**
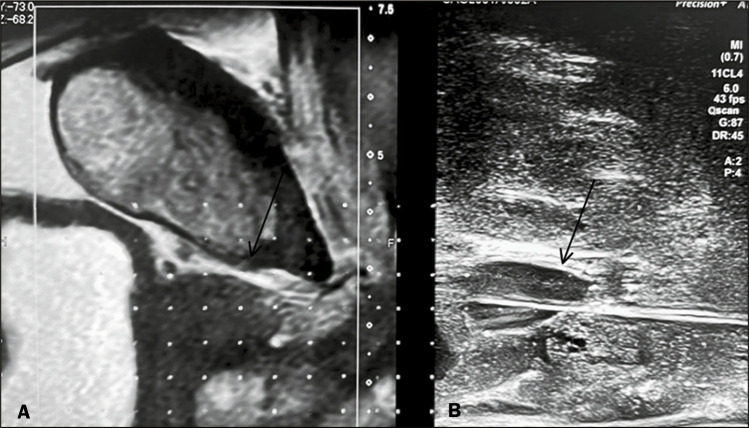
A: T2-weighted MRI showing atypical lymph node enlargement in the center of the image (arrow). B: Transperineal ultrasound with a 16-gauge biopsy needle in contact with the lymph node (arrow).

**Figure 6:**
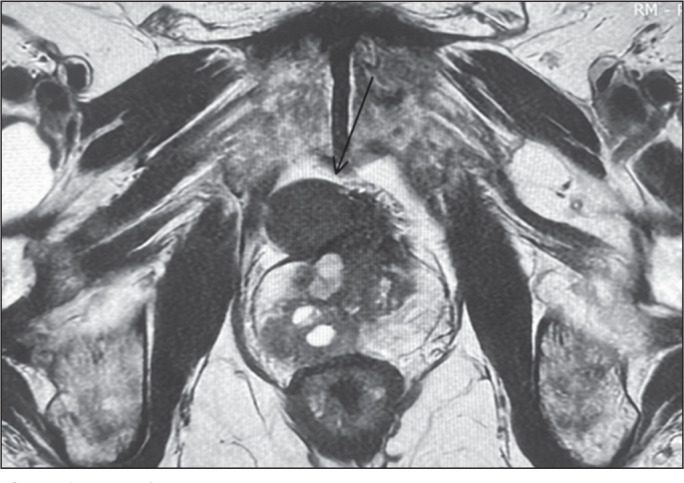
Axial T2-weighted MRI slice showing an atypical lymph node (arrow) in the pelvis.

**Figure 7:**
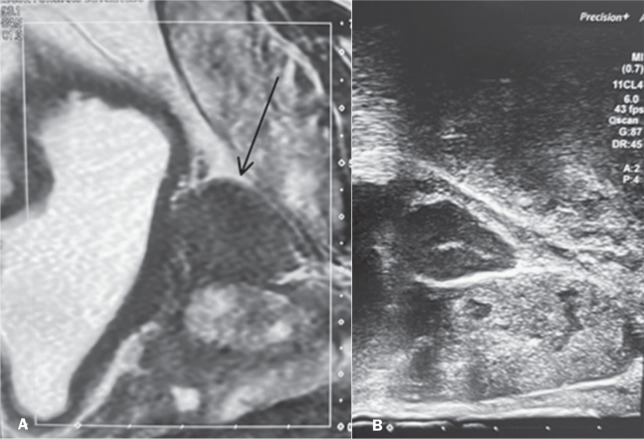
A: Sagittal T2-weighted MRI slice showing atypical lymph node enlargement. B: Ultrasound image showing the trajectory of the biopsy needle.

A 57-year-old male patient presented with atypical lymph node enlargement detected on MRI (Figures 5A, 6, and 7A). Ultrasound confirmed the location of the lymph nodes and guided transperineal access for biopsy with a 16-gauge needle, which allowed an accurate diagnosis (Figures 5B and 7B).

In the literature on ultrasound-guided transperineal access, there are few reports of biopsies of non-prostatic pelvic lesions, such as suspicious pelvic lymph nodes. Most such studies have focused on prostate biopsies. Nevertheless, there is evidence that this approach is effective, with a high success rate, depending on the size, location, and nature of the lesion, as well as on the experience of the practitioner. With transperineal access, complications such as bleeding and infection are rare. The approach is considered safe, especially when compared with methods that are more invasive, and is well tolerated by most patients.

### Placement of a spacer for radiotherapy

**Figure 8:**
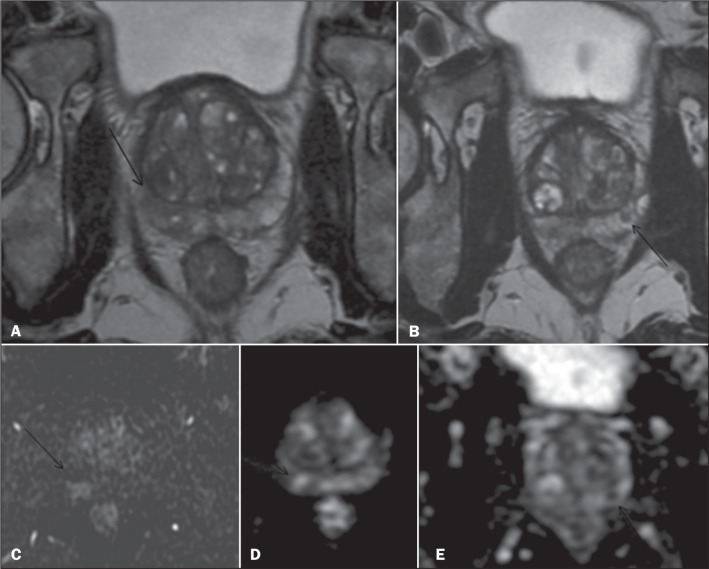
Nodular areas (arrows) with low signal intensity on T2-weighted imaging, showing moderately restricted diffusion on the right and markedly restricted diffusion on the left, consistent with PIRADS 4 lesions, later diagnosed as adenocarcinoma of the prostate.

**Figure 9:**
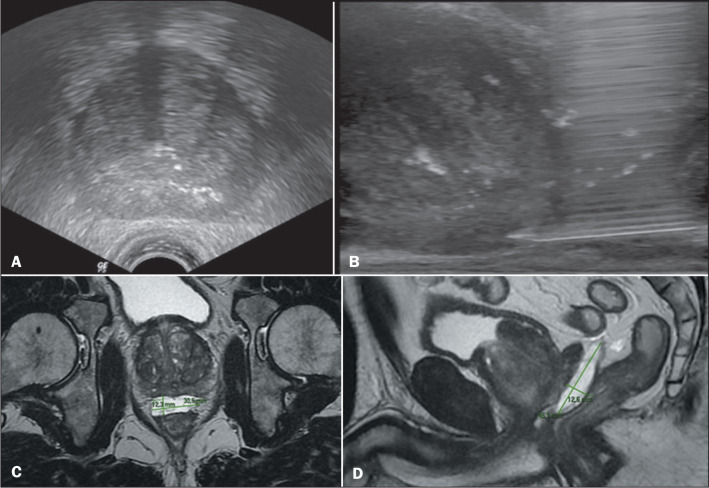
Procedure performed under ultrasound guidance (A,B), with technical success demonstrated on MRI scans (C,D).

A 73-year-old male patient declined to undergo surgery and, because of extensive bilateral (right-sided) disease (Figure 8), it was decided, together with the patient, to perform local treatment with radiotherapy for curative purposes. Therefore, the placement of a hydrogel spacer was proposed to minimize local, mainly rectal, side effects. The spacer was placed via ultrasound-guided transperineal access, and postprocedural imaging demonstrated technical success (Figure 9).

### Biopsy of perineal lesions

**Figure 10:**
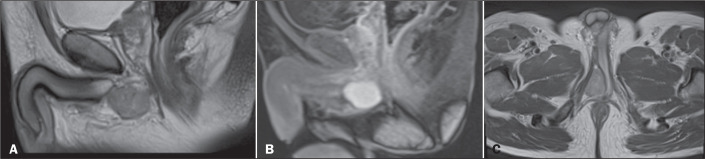
T2-weighted pelvic MRI scans showing a lobular, heterogeneous nodule with intense homogeneous enhancement in a patient diagnosed with liver neoplasia. Because of the location of the lesion, a transperineal approach was chosen for the biopsy, which resulted in the diagnosis of a neurofibroma.

A 48-year-old male patient was diagnosed with liver neoplasia and presented with a lobulated and heterogeneous nodule at the base of the penis. The nodule was visualized on MRI (Figure 10). Transperineal ultrasound confirmed the location of the lesion and guided the biopsy, allowing an accurate diagnosis. At this writing, the patient is being monitored by a multidisciplinary team and surgical excision of the lesion is being planned.

**Figure 11:**
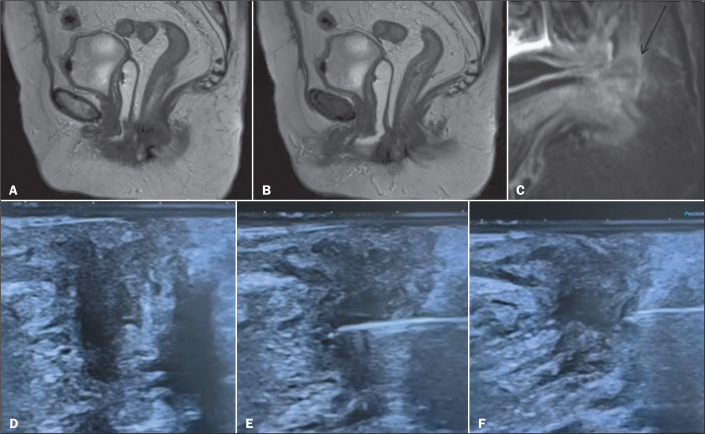
A-C: MRI scans showing an irregular lesion between the vagina and rectum. Transperineal biopsy was performed under ultrasound guidance (D-F).

A 62-year-old female patient presented with pelvic pain and intermittent vaginal bleeding. On MRI, an irregular mass was seen, located between the vagina and rectum (Figure 11A). The patient underwent a ultrasound-guided transperineal biopsy to confirm the diagnosis (Figures 11B and 11C). The pathology study of the biopsy sample resulted in a diagnosis of squamous cell carcinoma. On the basis of those findings, the treatment was planned, and, at this writing, the surgical planning is underway.

The literature on biopsies of perineal lesions performed via the transperineal approach is also quite limited. Nevertheless, this approach offers direct access to the lesions, minimizing the risk of complications and lesions in adjacent regions. Complications are rare, typically limited to mild bleeding and discomfort at the procedure site. Infections are also uncommon because of the “clean” path of the approach.

## LITERATURE REVIEW

The transperineal approach has been widely studied as an effective alternative route for various medical procedures. This approach has gained popularity because of its lower rate of infectious complications in comparison with transrectal and transvaginal approaches, as well as because it allows more direct access to specific structures of the pelvis. The most common procedures performed via the transperineal approach, together with their success rates and complications, are discussed below.

### Prostate biopsy

The most common procedure described in the literature for the transperineal approach is prostate biopsy. The transperineal approach is often preferred in patients at high risk of infection, such as those with prostatitis or a history of complications after transrectal biopsies. One comparative study showed that this approach has a significantly lower infection rate than the transrectal approach, with lower rates of infectious complications, as well as a lower incidence of fever and rectal bleeding. The diagnostic success rate of transperineal prostate biopsy is estimated to be greater than 95%, and it is more efficient in detecting cancer in anterior portions of the gland, which are less accessible in a transrectal approach. The main complications include acute urinary retention (in 5-10% of cases) and mild bleeding (hematuria and hematospermia). Serious infection, such as sepsis, is extremely rare, occurring in less than 1% of cases^**(11)**^.

### Prostate brachytherapy

Transperineal insertion of radioactive implants for the treatment of prostate cancer is a well-established method. This approach allows precise positioning of the material, typically iodine or palladium, within the prostate. Studies indicate that local tumor control is achieved in 80-90% of cases, depending on the stage of the disease. The most common complications include temporary urinary dysfunction and rectal irritation; serious complications, such as persistent urinary retention, are rare^**(12)**^.

### Treatment of pelvic abscesses

Drainage of pelvic abscesses, including prostatic and perirectal abscesses, can be safely performed via the transperineal approach under ultrasound guidance. The transperineal approach allows safe access without the need for procedures that are more invasive, such as open surgery. The literature reports complete resolution rates of 85-95%, with a good safety margin when compared with open surgery. The main complications include abscess recurrence (in 10-14% of cases) and, in rare cases, inadvertent injury to adjacent structures, which can be avoided with the use of image guidance^**(13,14)**^.

### Minimally invasive treatments for benign prostatic hyperplasia

Techniques such as water vapor thermal therapy (Rezum) or transperineal needle ablation are emerging as minimally invasive options for the treatment of benign prostatic hyperplasia in patients who are unwilling or unable to undergo traditional surgery. Studies show that such techniques provide significant improvement in obstructive urinary symptoms in 70-85% of patients, with a low risk of serious complications. Transient urinary symptoms such as dysuria and urinary urgency are common but usually resolve within weeks^**(15)**^.

In summary, the transperineal approach is considered safe and effective, although some complications can occur, such as infections, vascular or nerve injury, and perineal pain. Nevertheless, the infection rate is significantly lower than that associated with the transrectal approach, because the transperineal route avoids contact with the gastrointestinal tract. Serious infection, such as sepsis, is rare, occurring in less than 1% of cases. The risk of injury to perineal blood vessels and nerves is low, especially when the procedure is performed under guidance with imaging modalities such as ultrasound.

## CONCLUSION

This essay illustrates how transperineal ultrasound can be used for a wide variety of indications, standing out as a valuable tool for several clinical conditions, thus demonstrating remarkable versatility. The transperineal approach allows a more comprehensive exploration of the pelvic region, expanding its use beyond the diagnosis of prostate cancer.

The literature reports high rates of diagnostic and therapeutic success, together with a low incidence of serious complications, for procedures performed with a transperineal approach. The transperineal route stands out as a safe alternative, especially for patients at high risk for infection or in whom it is necessary to have direct access to pelvic structures that are difficult to reach by traditional routes.

For investigation of lesions in the pelvic region, careful selection of the technique is essential; the specific characteristics of the lesion and the individual needs of each patient should be taken into consideration. This essay underscores the importance of considering the transperineal approach as an important diagnostic and therapeutic option in various contexts. Advantages such as a reduced risk of infection, greater accuracy, and its minimally invasive nature make it a promising option. That increases the potential and clinical impact of the transperineal approach, making this multifaceted technique indispensable in modern medical practice.
